# What should be updated in the WHO guide to good prescribing?

**DOI:** 10.1007/s00228-026-04198-1

**Published:** 2026-10-14

**Authors:** Mariëlle G. Hartjes, Fleur M.W. Hendriksen, Michiel J. Bakkum, Yoann Cazaubon, Erik M. Donker, Ellen van Leeuwen, Robert Likic, Yves-Marie Pers, Tuan A. Phan, Joost D. Piët, Fabrizio De Ponti, Walter Raasch, Milan C. Richir, Floor van Rosse, Jitka Rychlíčková, Emilio J. Sanz, Markus Schwaninger, Susanna M. Wallerstedt, Michiel A. van Agtmael, Jelle Tichelaar

**Affiliations:** 1 Department of Internal Medicine, Amsterdam UMC, location Vrije Universiteit Amsterdam, unit Pharmacotherapy, De Boelelaan 1117, Amsterdam, 1081 HV The Netherlands; 2 Research and Expertise Centre in Pharmacotherapy Education (RECIPE), De Boelelaan 1117, Amsterdam, 1081 HV The Netherlands; 3 Interprofessional Collaboration and Medication Safety, Faculty of Health, Sports and Social Work, InHolland University of Applied Sciences, Pina Bauschplein 4, Amsterdam, 1095 PN The Netherlands; 4 Pathogenesis and Control of Chronic and Emerging Infections (PCCEI), University of Montpellier, INSERM, Avenue du Doyen Gaston Giraud, Montpellier, 34090 France; 5 Department of Fundamental and Applied Medical Sciences, Ghent University, C. Heymanslaan 10, Ghent, 9000 Belgium; 6 Unit of Clinical Pharmacology, Department of Internal Medicine, University Hospital Centre Zagreb and University of Zagreb School of Medicine, 12 Kišpatićeva St, 10 000, Zagreb, Croatia; 7 IRMB, University Montpellier, INSERM, CHU Montpellier, Montpellier, France; 8 Department of Hospital Pharmacy, Erasmus MC, University Medical Center Rotterdam, Rotterdam, The Netherlands; 9 Department of Medical and Surgical Sciences, Pharmacology Unit, Alma Mater Studiorum, University of Bologna, Via Irnerio 48, Bologna, 40126 Italy; 10 Institute of Experimental and Clinical Pharmacology and Toxicology, University of Lübeck, Lübeck, Germany; 11 Department of Surgery, University Medical Center Utrecht, Heidelberglaan 100, Utrecht, 3584 CX The Netherlands; 12 Department of Pharmacology, Faculty of Medicine, Masaryk University, Brno, Czech Republic; 13 School of Medicine, Universidad de La Laguna, and Hospital Universitario de Canarias (SCS), Santa Cruz de Tenerife, La Laguna, Tenerife 38071 Spain; 14 Department of Pharmacology, Sahlgrenska Academy, University of Gothenburg, Gothenburg, Sweden; 15 Centre for Health Technology Assessment, Sahlgrenska University Hospital, Region Västra Götaland, Gothenburg, Sweden

**Keywords:** Education, Medical education, Prescribing, Quality use of medicines

## Abstract

**Purpose:**

Over 30 years ago, the World Health Organization (WHO) published the Guide to Good Prescribing (GGP) with its accompanying six-step model for rational prescribing. While widely used and proven effective in teaching clinical pharmacology and therapeutics (CPT), the GGP has never been revised to reflect scientific, societal, technological, and educational developments. The aim of this study was to explore which elements could be revised to actualize its content.

**Methods:**

CPT teachers experienced with the GGP were recruited from all six WHO regions. Six online nominal group technique sessions were conducted to elicit and prioritize ideas for revising the GGP. Discussions were audio-recorded, transcribed verbatim, and analyzed using an inductive thematic approach to capture participants’ views on both content and optimal format of the renewed GGP.

**Results:**

Twenty-six participants shared136 ideas for the revision, grouped into three main areas: ideas about the usability, accessibility, and awareness of the GGP; ideas about the six-step framework; and ideas about background information or supplementary chapters. Suggestions for improving usability emphasized an interactive, digital platform for accessibility, continuous updates, and integrated resources. Regarding the six-step model, participants recommended retaining its structure while incorporating key considerations such as pharmacovigilance, shared decision-making, and deprescribing. Lastly, participants proposed supplementary information on topics such as therapeutic reasoning and special patient populations.

**Conclusion:**

Suggestions for revising the WHO GGP included integrating contemporary and societal prescribing considerations to enhance its relevance, educational impact and usability, thereby supporting safe and effective prescribing in modern clinical practice.

**Supplementary Information:**

The online version contains supplementary material available at https://doi.org/10.1007/s00228-026-04198-1.

## Introduction

**Fig. 1:**
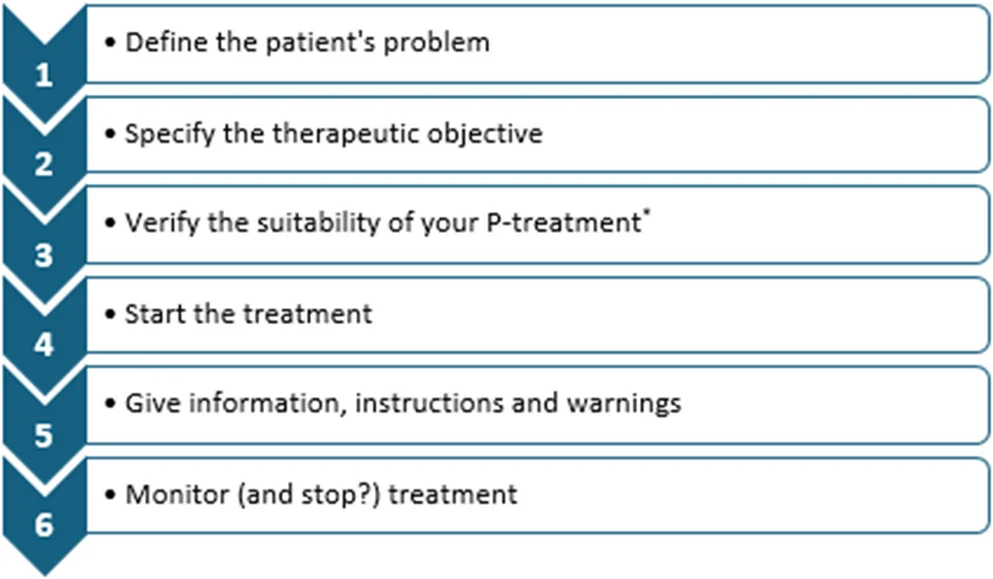
The 1993 WHO six-step model for therapeutic reasoning and rational prescribing * The GGP and six-step model use the principle of P-treatments and P-drugs,* in which prescribers choose a personal set of treatments and drugs for different indications*

Prescribing is an essential yet complex clinical skill in medical practice that should be developed effectively to optimize patient benefit while minimizing harm and medication errors [1, 2]. The World Health Organization (WHO) published the Guide to Good Prescribing (GGP) and its accompanying six-step model (Fig. 1) in 1993 as an educational tool to foster therapeutic reasoning and rational prescribing [3]. This was followed by the Teacher’s Guide to Good Prescribing (TGGP) in 2001, which helps clinical pharmacology and therapeutics (CPT) teachers to incorporate the GGP into their teaching [4].

In 2020, it was concluded in collaboration with the WHO that the GGP requires revision to remain fit for purpose as the healthcare context has changed over the last thirty years [5]. Although the need for revision was identified to ensure future relevance, recent systematic reviews indicate that the WHO six-step model is still widely used and is the most established approach for teaching rational prescribing [6], forming the foundation of many educational interventions [7–10]. Its structure is consistent with current theories on (context) learning, clinical decision making, and clinical practice [5, 7, 11].

However, medical practice has changed substantially since the GGP was published [5]. New treatments and guidelines have been introduced, digital support systems have become widely available, and more is appreciated about safeguarding planetary health and recognising the influence of the pharmaceutical industry. Educational methods have also evolved, with increasing emphasis on experiential and interprofessional learning [7, 12]. Many of these changes have been driven by technological progress, particularly the widespread availability of the internet, which allows rapid access to drug information and guidelines. In addition, societal perspectives have changed, including attitudes towards the doctor-patient relationship, social justice, sustainability and attention for diversity and inclusivity.

The GGP needs to be revised to ensure that it remains relevant and continues to support the educational needs of future prescribers in the context of these developments, as reported by Tichelaar et al. [5]. This can only be achieved with input from end-users and teachers. Therefore, the aim of this study was to explore and prioritize which adjustments users and educators deem most important for revision of the WHO GGP.

## Materials and methods

### Study design

This qualitative study used the Nominal Group Technique (NGT), a structured consensus method designed to generate and prioritize ideas from a group of individuals [13, 14]. NGT consists of structured focus group meetings five phases: (i) silent idea generation (ii), round-robin sharing and recording of the ideas (iii), group discussion about the presented ideas, aiming to clarify, combine, and/or remove ideas (iv), individual ranking of the ideas (v), sharing the ranked results, with optional further discussion. This structured format is designed to ensure equal participation, limit dominance, and facilitate systematic idea generation and prioritization [13, 14].

### Participants

Participants were recruited through three sources: (i) CPT teachers who registered on the project website to help with the revision of the WHO GGP, (ii) the WHO regional network, (iii) the Network of Teachers in Pharmacotherapy (NOTIP), which consists of over 400 CPT teachers from different European countries. We used purposive sampling to select participants to reflect a representation from all six WHO regions as shown in Table 1, and from both high-income and low-/middle-income countries. Participants were required to be familiar with the WHO GGP and to use it in their CPT teaching. Exclusion criteria were insufficient proficiency in English, as a shared language was necessary to ensure effective communication and consistency throughout the research process; however, this did not result in the exclusion of any participants. Additional exclusion criteria included conflicts of interest (e.g., financial ties to the pharmaceutical industry). Fifty-seven participants were invited to participate via email, with a reminder sent after 2 weeks. Invitations could be forwarded to qualified colleagues if relevant. Participants were informed about the study purpose and the researchers’ roles in pharmacotherapy education.

**Table 1:** Distribution of participants across the different WHO regions

WHO region	*N*	% Women	Median age (years)	Teaching experience (years, median)
Region of the Americas	2	50	47	16
European Region	13	46	56	23
African Region	2	0	36	8.5
Eastern Mediterranean Region	3	67	33.5	10
South-East Asia Region	2	50	38.5	8
Western Pacific Region	4	75	54	21

### Procedure

The meetings took place via Microsoft Teams and were recorded. Each meeting included three to six participants and three researchers (FH, MB, and JT), selected according to availability and WHO region for diversity where possible. Only participants and the research team were present during the meetings. Prior to the meeting, participants completed a questionnaire about their education, employment, and teaching experience. In preparation for the meeting, the participants (re)read the WHO GGP and the “WHO guide to good prescribing is 25 years old: quo vadis?” article [5]. Each meeting started with a round of introductions, followed by information about the study and the procedure during the meeting. The researchers introduced the topic and structured the meeting, asked for clarification when necessary, and summarized participants’ contributions to clarify the ideas, after which participants confirmed the accuracy of these summaries. The meeting continued with the five phases of the NGT. In the first phase, the participants were given 5 minutes to generate and organize their ideas. In the second phase, participants shared their ideas, which were recorded using the online platform Google Jamboard (no longer supported). In the third phase, the participants discussed the ideas and organized them into categories. Ideas that did not fit into a category remained separate. In the fourth phase, participants ranked their top five ideas or categories using Mentimeter (http://mentimeter.com), which generated an overall ranking subsequently verified by the participants in the fifth phase.

### Data analysis

Audio recordings were transcribed verbatim and analysed using an inductive thematic approach to capture participants’ explicit ideas and opinions regarding both the content to be included in the renewed GGP and the optimal format and delivery of the guide [15, 16]. For this analysis, two researchers (MH and FH) independently read the transcripts and generated a set of codes using MAXQDA (Standard 2022). Researchers proposed themes independently and then discussed them until consensus was reached. If no consensus was reached, a third researcher (JT) was consulted. The codes and themes were finalized and named in a meeting with the third researcher (JT). Participants did not provide feedback on the thematic analysis.

Items that were ranked first were awarded 5 points; items ranked second were awarded 4 points, etc. An average score per item was calculated to generate a final ranking of ideas for each meeting. If several items had the same average score, the number of participants that voted for the item was used to decide the rank of the items. This study is reported in accordance with the Consolidated Criteria for Reporting Qualitative Research (COREQ) 32-item checklist to ensure comprehensive and transparent reporting of qualitative methods and findings [17].

### Research team

MH is a woman, MD, PhD student and CPT teacher with 4 years’ research experience in CPT education and qualitative research. FH is a woman, medical student, and student coordinator of pharmacotherapy education with a master’s degree in child and education studies. MB is a man, MD, general practitioner and postdoctoral researcher in CPT education, with prior experience in NGT research and qualitative research. JT is a man, associate professor with over 20 years’ experience in pharmacotherapy education and is experienced in both qualitative research and NGT. The remaining authors are teachers in clinical pharmacology across Europe, including both women and men with MD or MScPharm qualifications. Researchers were actively involved in pharmacotherapy education, which may have influenced interpretation.

### Ethical aspects

The Medical Ethics Review Committee of the Amsterdam University Medical Center determined that the study did not fall within the scope of the Medical Research Involving Human Subjects Act (WMO) (reference: 2022.0274). Informed consent was obtained in advance from all participants.

## Results

In total, 26 participants from 20 countries across the six WHO regions participated in the study (Table 1), providing geographically diverse perspectives on the GGP and its use in education. Six meetings were organised and these lasted 55–105 min. The median age of participants was 48 years (range, 27–64 years), 50% was women, and they had 0.5–40 years (median 15.5 years) of teaching experience. In total, 136 adjustments were suggested in the six meetings. No new suggestions emerged in the last two meetings, at which point we decided that data saturation had been reached. The suggestions were discussed in the research team and categorized into three main themes as described below: ideas about the usability, accessibility, and awareness of the GGP; ideas about the six-step framework; and ideas about background information or supplementary chapters. The results of the ranking per meeting are shown in Table S1.

### Usability, accessibility, and awareness of the GGP

Multiple participants considered that the GGP should be updated to ensure its relevance for all prescribers (i.e. not only physicians, but also nurse-prescribers, dentists, midwives, and other non-medical prescribers) across a variety of healthcare settings including specialized and rural care, in both high-income and low-/middle- income countries, as demonstrated by the following quote:

“*And a lot of non-medical prescribers*,* certainly at the moment in the UK*,* are coming to prescribing training after qualifying as a healthcare professional. So*,* these different routes are going to affect*,* you know*,* your understanding of things. But I just think*,* if you want to get this to be engaged more widely*,* at the end of the day*,* good prescribing is good prescribing*,* regardless of who’s doing it.”* (P12).

The GGP should also be updated from its current PDF / Book form into a digital, interactive format with a concise core and expandable details and examples, as illustrated by one of the participants: “*Actually*,* what I mean by electronic book format is something like an app or an interactive format. It’s not just*,* you know*,* the reading and turning the page kind of electronic book*,* actually. And there might be some inputs and output to the system as well.”* (P1). This should be easily accessible via a dedicated webpage or application, preferably allowing offline accessibility in regions where there is limited internet connectivity. Additionally, this will allow future updates to be more readily implemented.

Participants recommended active promotion of the updated GGP for more widespread use, especially in regions where awareness of the GGP is currently lacking, as exemplified by the following quote: *“I think the guideline of good prescription is a very good guideline and education material for medical students and pharmacology students*,* but lastly*,* I think we need a dissemination plan of this good guideline. The dissemination plan*,* using technical developments*,* for example interactive online education or a periodic meeting*,* especially for low income countries.” *(P16). Possible ways to improve dissemination are through dedicated teach-the-teacher courses (such as the events held in the early days of the GGP, although preferably online) and via existing (online) teacher networks and platforms such as the NOTIP network, International Union of Basic And Clinical Pharmacology Education section (IUHPAR ED), and the European Open Platform for Prescribing Education [18–20]. In addition, to ensure optimal use, the effectiveness of the updated guide should be evaluated in research studies.

### The six-step prescribing framework

Participants considered the six-step framework well known and educationally effective, and therefore advocated retaining it as the core structure of the GGP, as shown in the following citation: *“First of all*,* I would not change the steps because to make it as simple as possible*,* I literally love the six steps. We can refine the steps.”* (P6). However, rather than six linear steps, the framework should be updated to emphasize the circular, iterative process of safe and effective prescribing.

*Step 1: Define the patient’s problem & step 2: Specify the therapeutic objective*.

The updated GGP should promote a holistic view of the patient. Users should be educated that the therapeutic objective is rarely as simple as treating an illness, but may vary dependent on patient background, behaviour, comorbidities, and importantly the patient’s own ideas, concerns and expectations. The importance of adequate history taking and obtaining an accurate medication list should be emphasized.

#### Step 3a: choose your standard treatment

The GGP should place greater emphasis on clinical/therapeutic reasoning rather than focusing primarily on the selection of a personal standard treatment. While some participants felt that guidance on developing and maintaining a personal formulary remains relevant, others felt that the guide should acknowledge that prescribing is increasingly guideline oriented. Consequently, more guidance should be provided on how to use clinical guidelines. In addition to this, it is important that users understand the reasoning behind the guidelines and are be able to critically reflect on this, as exemplified by the following quote: *“(…)*,* because I think prescribers can rely too much on guidelines and trial evidence*,* without thinking back to what they know about the basic pharmacology and clinical pharmacology that would actually be relevant to whether this patient is suitable for this drug or not.”* (P20). There was no consensus about the use of P-drugs (personal formularies) in this study. It was stated that when P-drugs are used as part of this process, they should be tailored not only to the prescriber, but also to individual patients or specific patient groups (e.g. older adults, paediatric or pregnant patients). Additionally, the impact of medicines on planetary health should be weighed and several participants stated that where applicable, non-pharmacological alternatives may be considered.

#### Step 3b: verify the suitability of your treatment

The update should provide guidance on new factors to consider in selecting the most suitable treatment plan. Pharmacogenomics are an increasingly relevant factor to consider in daily practice, as is the restricted availability of medicines, which affects treatment choices. Lastly, shared decision-making has assumed a more prominent role compared to 1993 and when possible, treatment decisions should be made in consultation with the patient.

#### Step 4: start treatment

In the current version of the GGP, this step is too focused on writing a prescription on paper, while prescribing is also often done in an electronic patient record in many settings nowadays, which may lead to other challenges. These electronic prescribing systems often provide some form of decision support, and the update should guide users on the advantages and dangers (e.g. alert fatigue, undesirable defaults, automatic prescribing) of such systems. The update should emphasize the relevance of documenting in the medical record the reasoning behind a treatment choice and important monitoring measures. One participant explained: *“I think that’s actually something that’s missing from this (…)*,* but it’s critically important [to] document the decision. And including not just what you’ve done*,* but why.”* (P20).

#### Step 5: give information, instructions, and warnings

Because of the internet, patients are more knowledgeable and opinionated about their treatment preferences than when the GGP was first published. Therefore, the update should provide guidance on patient communication. This should include dealing with patients who are influenced by medical misinformation spread via social media, which is a growing problem according to the participants. Additionally, guidance should be provided on communication about treatment adherence and potential adverse effects, ensuring that patients receive adequate information and give their informed agreement to treatment, while avoiding overloading them, which could lead to nocebo effects. As stated by one of the participants: *“For instance*,* due to cultural differences or low income or language barriers*,* all those areas where lots of people*,* well*,* there are problems with taking medication*,* understanding why they’re not taking the medication is important. And maybe also some solutions in the guide on how to deal with that.”* (P13).

#### Step 6: monitor (and stop? ) treatment

The update should focus not only on monitoring and discontinuing the given treatment, but also on adjusting and re-evaluating other ongoing therapies through medication review and deprescribing. Considerations should include stopping medication with little or no benefit in frail and/or older patients, and considering the environmental impact of the patient’s current medicines (e.g. switching metered dose inhalers to dry-powder ones) [21]. These topics are interlinked, as demonstrated by the following citation: *“When you talk about sustainability*,* we mentioned do not waste drugs. Maybe just another example… Deprescribing*,* and*,* which is step 6*,* how do I monitor and perhaps stop treatment? So deprescribing is part of sustainability.”* (P6). Further guidance should be provided on how to cope with adverse effects and mitigating prescribing cascades.

### Background information or supplementary chapters

The participants also felt that the GGP should provide background information on societal and educational developments, in addition to updates on these subjects in the six-step framework. Societal developments include increased attention for inclusion, diversity, and equity (including intercultural differences in prescribing), planetary health, coping with for example workplace pressure to avoid prescribing errors, and the influence of the pharmaceutical industry. Educationally, the update should provide a theoretical basis for therapeutic reasoning and information on how to use guidelines wisely, personal formularies (p-drugs), and making efficient use of and critically evaluating trustability of new sources of information (including artificial intelligence).

Additionally, the participants suggested that the update would benefit from foundational background information on the following subjects: legal aspects of prescribing; prescribing under specific circumstances, such as during a virtual consult or during a pandemic; prescribing for specific populations (e.g. older adults, paediatric and pregnant patients, and those with chronic kidney disease, liver disease or obesity). This is captured by the following quote: *“In some countries*,* including Korea*,* there has been a rapidly changing patient population over 25 years. For example*,* in Korea*,* the elderly population is increasing rapidly. So*,* the drug safety issue*,* including polypharmacy*,* is very important.”* (P16).

Other topics include principles of pharmacokinetics and therapeutic drug monitoring; prescribing high-risk (e.g. antimicrobials, opioids, biologicals, and corticosteroids) or particularly newsworthy drug classes (e.g. anti-obesity drugs); and the practicalities of prescribing, such as calculating drug dosages, handling repeat prescriptions, working in interprofessional teams, and drug administration.

## Discussion

This study identified educators’ suggestions for revising the WHO GGP and its six-step model and classified them into three overarching themes: a digital, open format; an updated six-step framework that reflects contemporary practice; and supplementary content on emerging topics, such as describing and planetary health.

A key outcome relates to the format of the revised GGP. Participants emphasized the need for an online, interactive platform to improve accessibility, enable continuous updates, and allow internal linking to related sections and external resources. This preference is supported by evidence that digital health education is at least as effective as traditional methods, also within prescribing education [22–25]. Suggested design features, such as concise sections with expandable details, multimedia elements, and case-based simulations, are consistent with known principles of digital learning [23]. Existing networks for pharmacotherapy teachers, such as the NOTIP network, which is affiliated with the Education Working Group of the European Association for Clinical Pharmacology and Therapeutics (EACPT), the IUPHAR ED and the European Open Platform for Prescribing Education, may be leveraged to improve dissemination and uptake [18–20]. To improve usability, the updated guide and its contents should be free of costs and copyright restrictions, making it effectively an open educational resource. For example, a digital GGP could allow learners to work through a prescribing case, receive step-specific prompts aligned with the six-step model, link directly to local formularies and guidelines, and document therapeutic reasoning in a structured way, thereby reinforcing reflective prescribing practice at the point of care.

Participants agreed that the six-step model should remain the foundation of the GGP, but proposed incorporating several additional aspects that have gained importance over the past three decades. These include topics such as planetary health and deprescribing, which reflect broader societal developments. Knowledge about these subjects has increased substantially over the past thirty years, which could explain why participants suggested them and why they were not previously included in the GGP. For example, growing evidence documents the bidirectional relationship between climate change and health [26], and the environmental footprint of medicines has become a recognized concern [27]. Similarly, the increasing burden of polypharmacy highlights the need for medication reviews and deprescribing, including specifying a stop date for each medication and, when appropriate, a tapering schedule, as well as preventing the continued use of medications beyond their recommended duration [28–30]. Incorporating these topics supports the development of prescribers’ therapeutic reasoning skills while ensuring that the guide remains person-centred and responsive to global challenges. The proposed updates to the GGP map closely onto contemporary competency frameworks for prescribing (e.g. person-centered care, shared decision-making, deprescribing, digital literacy). Explicitly aligning the revised GGP with competency-based curricula could facilitate integration into undergraduate and postgraduate training and support assessment of prescribing competence across contexts.

A third key theme was the request for supplementary information. Participants proposed a wide range of additional topics, from general guidance on therapeutic reasoning and antibiotic stewardship to detailed advice for specific patient populations, underscoring the complexity of prescribing and highlighting the need for a comprehensive, integrated resource that can support prescribers across diverse clinical and contextual settings. Integrating therapeutic reasoning explicitly ensures that users understand why certain decisions are made, which will increase their ability to apply knowledge flexibly and safely, and facilitate the transfer of these skills to novel clinical scenarios. By including these topics, the guide can help users apply therapeutic reasoning in a safe, evidence-based, and patient-centred manner, while remaining aware of the technological and regulatory context in which they practice. However, the participants’ request for more core-medical subjects, such as opioid prescribing or pharmacokinetics, may be beyond the scope of the planned update. However, where applicable weblinks to credible and openly accessible resources may provide a solid alternative for the users and these topics might be addressed within case examples throughout the revised guide. While the six-step model offers a universal cognitive scaffold for prescribing, participants emphasized that implementation must remain locally adaptable (e.g. medicine availability, healthcare system constraints, digital infrastructure). A modular digital GGP could reconcile global principles with local practice through region-specific layers or plug-ins. Particular attention should be paid to the applicability of the revised GGP in low- and middle-income countries, although not all recommendations will be equally relevant across all healthcare settings. Similar contextual differences already existed during the development of the original guide in the 1993, reflecting variation in healthcare systems, medicine availability, and educational infrastructure. Nevertheless, the revised GGP should aim to provide a universally relevant framework for rational prescribing, while allowing sufficient flexibility for adaptation to local contexts and needs.

Importantly, many of the topics proposed by participants are covered in the existing literature, indicating that their suggestions reflect established and emerging challenges in prescribing practice. For example, the emphasis on formulating clear therapeutic goals has previously been shown to be effective, for example according to SMART principles [31]. Likewise, prescribing cascades and alert fatigue are increasingly recognized as contributors to inappropriate prescribing and medication-related harm [32–35]. The attention to planetary health further reflects the growing international discourse on sustainable prescribing [27, 36].

These findings highlight the evolving scope of rational prescribing and the need for the GGP to balance comprehensiveness with usability. Expanding the guide to cover contemporary priorities must be matched with careful attention for accessibility, practicality, and global relevance. Thoughtful integration of digital tools, interactive learning, and context-sensitive content will be essential to ensure that the revised GGP remains relevant, widely applicable, and effective in supporting prescribers worldwide. In addition, emerging technologies such as artificial intelligence are likely to play an increasingly prominent role in healthcare and prescribing practices in the coming years, and its role should therefore also be considered in the future development of the GGP [37]. However, further research is needed to understand how AI can be effectively and safely integrated into therapeutic decision-making.

### Strengths and limitations

This study has several strengths and limitations. A key strength is that this is the first study in thirty years to systematically collect and prioritise ideas for updating the WHO six-step from CPT teachers who actively use the six-step in their CPT education. Another strength lies in the methodology used. NGT facilitated comprehensive idea generation with attention for equal input from all participants, and the ranking helped us to prioritize the ideas [13, 14]. NGT was recently proven to be effective in CPT education research [38]. The inclusion of participants from all WHO regions provided a diverse range of perspectives and backgrounds, encompassing professional, academic, and educational experiences. Despite this, the international composition of the groups also posed challenges. National and international guidelines are not always uniform, which sometimes led to contradictions in the discussions that had to be addressed individually. Although invitations were distributed broadly across regions, most responses came from Europe. This may partly reflect the presence of well-established professional and educational networks in Europe, whereas such networks may be less developed or less accessible in other regions. Consequently, representation across regions was uneven, although participants from all WHO regions were included. These are the formal WHO regions, so perspectives are representative at the regional level, though subregional variation may exist and may therefore impact generalizability.

Conducting the NGT online allowed the inclusion of participants from different regions, but this format could have influenced interaction.However, previous research has shown that conducting NGT meetings online is viable with careful preparation [39]. Our purposive sampling led to the inclusion of predominantly known associates and experienced teachers, possibly leading to an underrepresentation of less-experienced or less-connected teachers and we were unable to include non-English speakers. Although NGT aims to minimize dominance, subtle power dynamics related to seniority, academic reputation, or regional representation may still have influenced the prioritization of ideas. Finally, the proposed digital, adaptable format of the updated GGP allows for continuous updates, in which we aim to include the voice of previously unheard colleagues.

### Conclusion

In conclusion, this study provides an overview of the adjustments needed for a contemporary revision of the WHO GGP. Input from educators across all WHO regions showed support for preserving the six-step model while expanding its scope to reflect today’s clinical realities, including person-centred care, deprescribing, digital decision support, and planetary health. Participants also highlighted the importance of a flexible digital format that allows continuous updates, wider dissemination, and integration of supplementary learning resources. Incorporating these priorities will improve the relevance, usability, and global applicability of the revised GGP. A modernized digitally accessible guide has the potential to strengthen prescribing competence, support harmonization of CPT, and ultimately promote more rational use of medicines worldwide. Based on this research, the next step is the revision of the GGP which will follow in the upcoming years. 

## Electronic Supplementary Material

Below is the link to the electronic supplementary material.

Supplementary Material 1 (DOCX 29.7 KB)

## Data Availability

The data that support the findings of this study are available from the corresponding author upon reasonable request.
