# Supplementary material for: What should be updated in the WHO guide to good prescribing?
Source: Eur J Clin Pharmacol. 2026 Oct 14;82(11):270. doi: 10.1007/s00228-026-04198-1 (PMC13668889; doi:10.1007/s00228-026-04198-1)
Supplement: Supplementary file 1 [file 228_2026_4198_MOESM1_ESM.docx]

# S1: Nominal group results

| **Meeting 1** (five participants from Belgium, Peru, Turkey (2 participants), and Uruguay) | | | | |
| --- | --- | --- | --- | --- |
| **Rank** | **Idea** | **Average score*** | **Number of votes** | |
| 1 | Comprehensive medication approach (review, deprescribing, polypharmacy, repeat prescriptions) | 4.5 | 4 | |
| 2 | Emphasis on identifying the therapeutic objective | 3.67 | 3 | |
| 3 | Emphasize need for critical thinking (application of literature, evidence-based medicine) | 3.33 | 3 | |
| 4 | Future proof format (rich, interactive, digital, convincing) | 3.0 | 5 | |
| 5 | Local availability of medicines | 3.0 | 1 | |
| 5 | Define the target population of the guide | 3.0 | 1 | |
| 6 | Patient empowerment (how to convince patients, improve adherence, promotion of health literacy) | 2.0 | 5 | |
| 7 | Recommendation to use digital support systems | 2.0 | 2 | |
| 8 | (Clinical) pharmacologists should be in charge of teaching pharmacotherapy ** | 1.0 | 1 | |
| **Meeting 2** (four participants from India, Iran, Italy, and Romania) | | | | |
| **Rank** | **Idea** | **Average score*** | **Number of votes** | |
| 1 | Novel teaching ideas ** | 5.0 | 1 | |
| 1 | Guidelines specific to geriatric patients (DDIs, polypharmacy, etc.) | 5.0 | 1 | |
| 2 | Six-step should remain (well-known concept) | 4.5 | 2 | |
| 3 | Treatment during pandemics | 4.0 | 1 | |
| 4 | Inclusivity (of all situations and patient contexts, e.g. remote areas with few possibilities for monitoring) | 3.5 | 2 | |
| 5 | Sustainability (deprescribing, antimicrobial stewardship) | 3.0 | 2 | |
| 6 | Create network / Teach the teacher ** | 2.75 | 4 | |
| 7 | More focus on patient safety (communication, pharmacovigilance) | 2.67 | 3 | |
| 8 | Use of digital information sources | 1.33 | 3 | |
| **Meeting 3** (five participants from Albania, Netherlands (two participants), Spain, and United Kingdom) | | | | |
| **Rank** | **Idea** | **Average score*** | **Number of votes** | |
| 1 | Medication review (deprescribing, prescribing errors, omissions) | 4.4 | 5 | |
| 2 | Form of the guide | 3.75 | 4 | |
| 3 | (New) influences on decision making | 2.8 | 5 | |
| 4 | Adverse drug reactions | 2.75 | 4 | |
| 5 | It should be a guide for all prescribers, not just medical | 2.33 | 3 | |
| 6 | More focus on history taking | 2.0 | 1 | |
| 7 | Shared decision making | 1.5 | 2 | |
| 8 | Focus on health literacy | 1.0 | 1 | |
| **Meeting 4** (five participants from Australia, Egypt, South Africa (two partipants), and South Korea) | | | | |
| **Rank** | **Idea** | **Average score*** | **Number of votes** | |
| 1 | Safe drug use and avoiding abuse (opioids, corticosteroids, hormonal supplements, antimicrobial stewardship, therapeutic drug monitoring) | 4.0 | 5 | |
| 2 | Digital transformation of the guide(s) | 3.8 | 5 | |
| 3 | Electronic prescribing | 3.5 | 2 | |
| 4 | Benchmark global training ** | 3.0 | 4 | |
| 5 | Context, special groups, and special situations | 2.67 | 3 | |
| 6 | Modernized drug info resources, Cochrane reviews | 2.0 | 1 | |
| 6 | Appendix on infusion rates and other pharmacological calculations | 2.0 | 1 | |
| 7 | Patient communication, patient education and adherence | 1.25 | 4 | |
| **Meeting 5** (three participants from Australia, Czech Republic, and New Zealand) | | | | |
| **Rank** | **Idea** | **Average score*** | **Number of votes** | |
| 1 | Re-evaluating the concept of P-drugs | 4.0 | 3 | |
| 2 | Links to tools (e.g. for calculation and communication) | 4.0 | 1 | |
| 3 | Section on personalized medicine (including pharmacogenomics/-genetics) | 3.0 | 2 | |
| 4 | More holistic approach to step 1 (drug, patient and disease characteristics) | 2.67 | 3 | |
| 4 | Shared decision-making and patient communication | 2.67 | 3 | |
| 5 | Documentation | 2.33 | 3 | |
| **Meeting 6** (four participants from Bulgaria, Egypt, Germany, and India) | | | | |
| **Rank** | **Idea** | **Average score*** | **Number of votes** | |
| 1 | Suggest non-pharmacological approach whenever possible (minimize drug use) | 5.0 | 1 | |
| 2 | Use up to date guidelines for P-drugs / selecting the right drug | 3.5 | 4 | |
| 3 | Distinction between primary and secondary care in updated case-vignettes and teaching examples | 3.25 | 4 | |
| 4 | Essential drugs | 3.0 | 4 | |
| 5 | Emphasis on clear history taking and information gathering | 2.67 | 3 | |
| 6 | Emphasis on patient education (to improve drug adherence) | 2.0 | 4 | |
|  | | ** out of 5* | |  |

** These ideas were related to the Teacher’s Guide to Good Prescribing. Since the focus of this study was the Guide to Good Prescribing itself, these ideas were not included in the article.
